# Using protein turnover to expand the applications of transcriptomics

**DOI:** 10.1038/s41598-021-83886-7

**Published:** 2021-02-23

**Authors:** Marissa A. Smail, James K. Reigle, Robert E. McCullumsmith

**Affiliations:** 1 Department of Pharmacology and Systems Physiology, University of Cincinnati, 2170 E. Galbraith Rd. Bldg E. Room 216, Cincinnati, OH 45237-0506 USA; 2 Neuroscience Graduate Program, University of Cincinnati, Cincinnati, OH USA; 3 Biomedical Informatics, Cincinnati Children’s Hospital Medical Center, Cincinnati, OH USA; 4 Department of Neurosciences, University of Toledo College of Medicine and Life Sciences, Toledo, OH USA; 5 Neurosciences Institute, ProMedica, Toledo, OH USA

**Keywords:** Computational biology and bioinformatics, Molecular biology, Neuroscience

## Abstract

RNA expression and protein abundance are often at odds when measured in parallel, raising questions about the functional implications of transcriptomics data. Here, we present the concept of persistence, which attempts to address this challenge by combining protein half-life data with RNA expression into a single metric that approximates protein abundance. The longer a protein’s half-life, the more influence it can have on its surroundings. This data offers a valuable opportunity to gain deeper insight into the functional meaning of transcriptome changes. We demonstrate the application of persistence using schizophrenia (SCZ) datasets, where it greatly improved our ability to predict protein abundance from RNA expression. Furthermore, this approach successfully identified persistent genes and pathways known to have impactful changes in SCZ. These results suggest that persistence is a valuable metric for improving the functional insight offered by transcriptomics data, and extended application of this concept could advance numerous research fields.

## Introduction

The use of RNA sequencing (RNAseq) to assess global changes in gene expression has grown rapidly in the last decade^1–3^. RNAseq allows researchers to collect transcriptomic signatures in their tissue and disease of study, yielding extensively more data than more traditional methods such as qPCR. This comprehensive view of the molecular landscape has been pivotal to uncovering novel disease mechanisms and targets. As the cost of RNAseq drops and the technology improves, it will be a central tool in biological studies moving forward^3–5^.

While RNAseq is a powerful tool, it has one major caveat, in that RNA expression does not necessarily match protein abundance. RNA may be present in a cell, but we have no way of knowing the rate at which that RNA is translated into protein or if that protein is active in the cell. This disequilibrium between RNA and protein suggests that RNAseq results could be misleading and fail to reveal functional insights into cellular activity and disease mechanisms^5–8^. While proteomics is an option, RNAseq is more commonly used and cost-effective, so finding a way to extend RNAseq applications to gain insight into protein abundance would allow use of existing mRNA datasets to gain a deeper understanding of diseases or model systems^8^.

Here, we propose a novel method of using protein turnover ratios to infer protein abundance from RNAseq and microarray data, thereby extending its functional value. Every protein has a particular half-life, and therefore exists in a cell to exert its functions for a relatively predictable amount of time^9,10^. We propose that using this information about protein half-life in tandem with transcriptomics data could offer insights into protein abundance. Proteins that are degraded quickly may show low protein abundance even if there is a large amount of RNA present, and proteins that last may show high protein abundance even if there is lower RNA. We coined the term “persistence” to define this relationship between RNA expression, protein half-life, and protein abundance, and propose that analyzing persistence could offer greater functional insights into traditional RNAseq datasets^11,12^.

We utilized existing protein turnover data in combination with schizophrenia (SCZ) RNAseq and RNA microarrays to assess this concept and demonstrate its potential application in disease states. In a 2018 paper, stable isotope labeling in mammals (SILAM) was used to assess protein turnover in mouse synaptosomes, and turnover ratios were determined for ~ 2200 proteins in the brain^13^. We used these data to establish persistence scores and applied these values to existing RNA datasets. Three existing RNA datasets from our lab containing control (CTL) and SCZ subjects were examined. Here we assessed (1) the relationship between RNA expression and protein turnover ratios in CTL and SCZ; (2) the genes which exhibit high or low persistence in SCZ; (3) the biological functions associated with high and low persistence; and (4) the relationship between persistence and protein abundance.

While our results show that there is not a direct relationship between turnover and RNA expression or a shift in persistence in SCZ, genes identified as high and low persistence have previously been implicated in the functional deficits of SCZ. Additionally, our current method improves the ability of RNA data to predict protein data, and further studies with larger datasets and machine learning could be used to further improve the persistence calculation. More precisely identifying the relationship between RNA, protein, and turnover stands to greatly expand the applications of transcriptomics by allowing it to offer insight into protein function.

## Results

### There is not a relationship between protein turnover ratios and RNA expression

**Figure 1:**
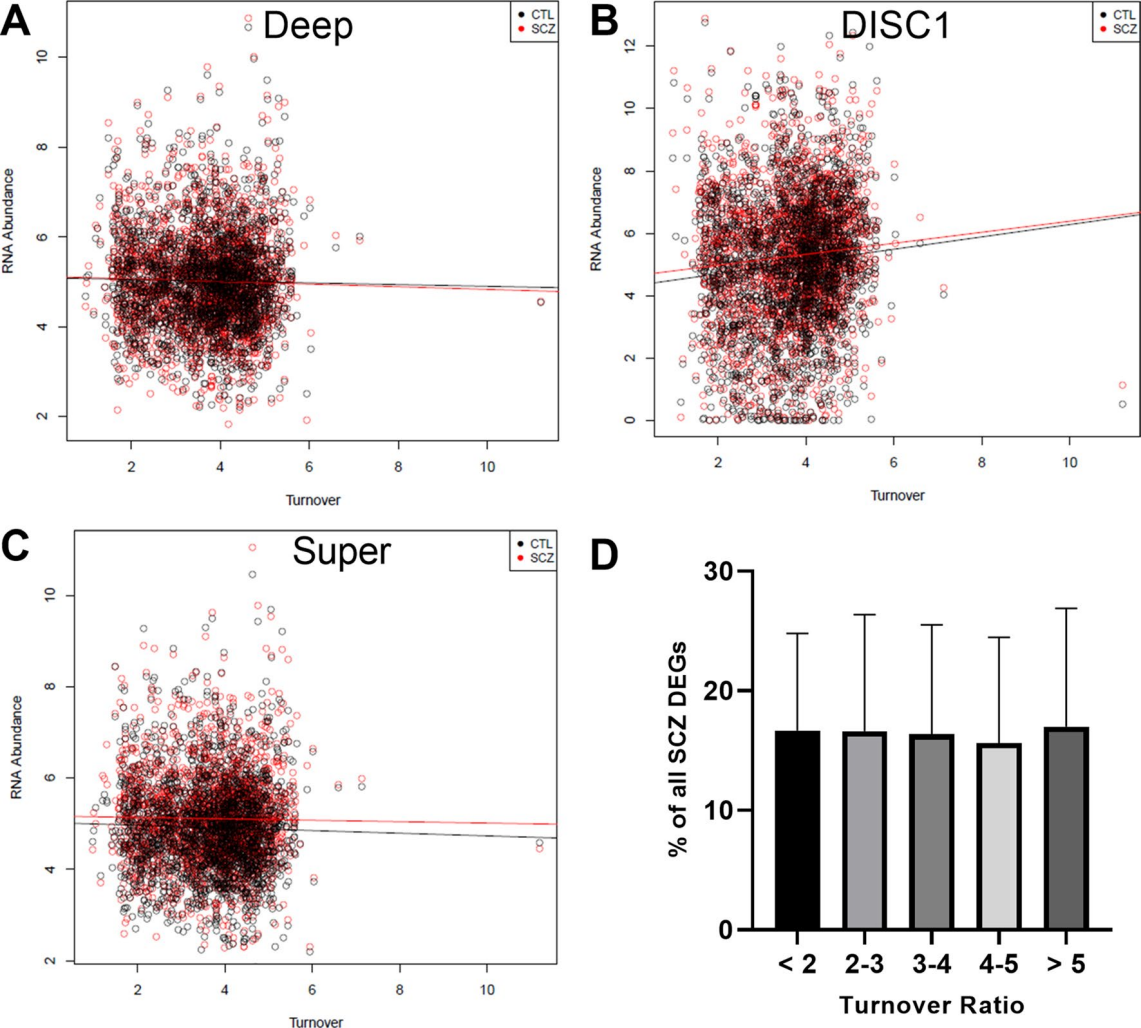
No clear relationship between protein turnover and RNA expression, in control or disease states. (**A**–**C**) Correlation plots between protein turnover ratios and RNA expression in (**A**) Deep, (**B**) DISC1, and (**C**) Super datasets. No relationships were identified in CTL (black dots) or SCZ (red dots) subjects. Figures produced using R base graphics^45^. (**D**) No shift in gene fold change in SCZ as a function of turnover ratio. Differentially expressed genes (DEGs) in different turnover ratio bins showed similar degrees of expression changes between SCZ and CTL samples across all 3 datasets, suggesting that no particular bin was especially sensitive to SCZ disease effects. Figures produced using GraphPad Prism 8^46^.

Prior to moving forward with the idea of persistence, we wanted to determine if a simpler way to connect RNA and protein expression existed. We began by assessing the base relationship between protein turnover and RNA expression with the idea that perhaps proteins that turnover faster are transcribed faster or vice versa. If this were the case, it would suggest that RNA expression and protein turnover balance out to support a more direct relationship between RNA and protein. However, regression analysis revealed that there is no relationship between turnover and RNA in CTL or SCZ in any of our datasets (Deep CTL: Adj R^2^ = 0.000135, Deep SCZ: Adj R^2^ = 0.000267, DISC1 CTL: Adj R^2^ = 0.000843, DISC1 SCZ: Adj R^2^ = 0.000672, Super CTL: Adj R^2^ = 0.000319, Super SCZ: Adj R^2^ = -0.000231) (Fig. 1A–C). This suggests that there is a more complex relationship between RNA and protein across the transcriptome. While this is not surprising given the literature, it confirms that we cannot simply assume that high turnover proteins are transcribed more, and that a more complex calculation is needed to integrate these measurements in a way that is informative.

### SCZ does not shift RNA expression in high or low turnover proteins

While no overall relationship was observed between turnover and RNA, it is possible that disease states could have exaggerated effects on certain subsets of protein, based on their turnover. For example, short-lived proteins could be expressed more in SCZ than long-lived proteins or vice versa. In order to look for this specific enrichment in disease, we broke the turnover ratio data into 5 bins ranging from very high turnover (quick half-life) to low turnover (long half-life). We then examined how many genes in each bin were differentially expressed (DEGs) in all three of our SCZ datasets and normalized this to the number of genes in the bin, yielding the % of DEGs in each bin (Fig. 1D). A one-way ANOVA (F(4,10) = 0.003; *p* > 0.999) revealed that there was no particular enrichment in any one bin, suggesting that SCZ does not specifically enrich genes based on turnover ratio. This shows that not only is there no particular vulnerability of proteins to SCZ as a function of their turnover, but also that disease effects on turnover will not bias the following persistence calculations.

### High and low persistence genes correspond to pathways known to be impacted by SCZ

**Figure 2:**
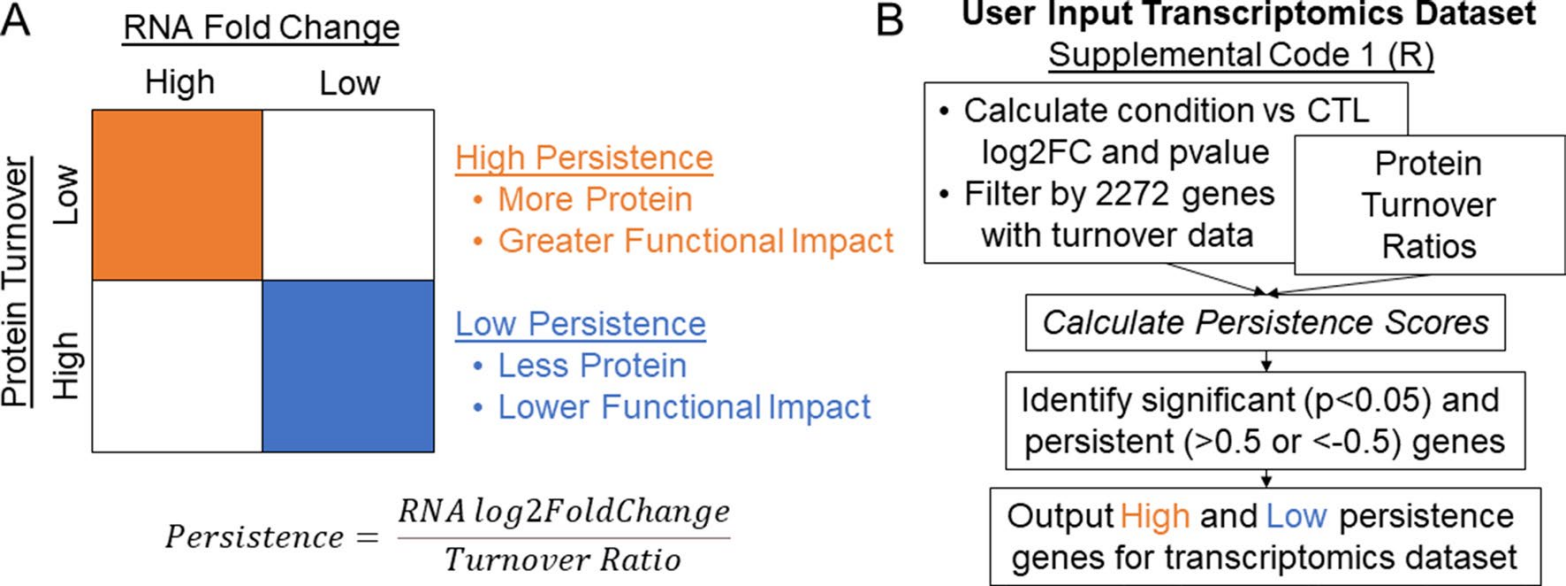
Schematic of persistence concept and analysis. (**A**) Conceptual framework for persistence, which considers protein turnover and RNA fold change together to gain a deeper understanding of the functional impact of changes in gene expression by estimating changes in protein expression. High persistence genes (orange) are thought to have a greater functional impact because they are produced more (high RNA expression) and remain in the cell longer (low protein turnover). Low persistence genes (blue) are thought to have a lesser functional impact because they are not produced as much (low RNA expression) and are cleared from the cell faster (high protein turnover). In this way, the persistence formula considers protein turnover and RNA fold change together and generates persistence scores provide deeper functional insights into RNA changes by acting as a proxy for protein changes. (**B**) Outline of code used to produce persistence scores and identify genes of high or low persistence. See Supplemental Code 1 for more details.

**Figure 3:**
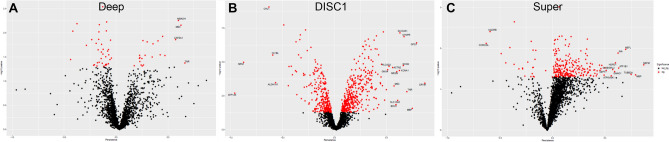
Persistent, significant genes in SCZ. Volcano plots of persistence scores and − log10 *p* values from (**A**) Deep, (**B**) DISC1, and (C) Super datasets. Red dots signify significant (*p* < 0.05) genes, and labels signify genes that also surpassed the persistence cutoff of 0.5. These genes were assigned as having low (< − 0.5) or high (> 0.5) persistence and used in subsequent analyses. See Table 1 for a full list of persistent genes. Figures produced using ggplot2^48^.

In the absence of direct relationships between RNA and protein or predictable disease effects, we developed the concept of persistence. Figure 2 provides a conceptual overview of persistence, as well as an outline for Supplemental Code 1 which can be used to calculate persistence for any input transcriptomics datasets. In the present analysis, turnover ratios were available for 2272 genes^13^. Of these, 2101 were found in our 3 RNA expression datasets, so persistence scores were calculated for these genes (Eq. (1) and Supplemental Table 1). The overall relationship between persistence and p value in our 3 SCZ datasets can be found in Fig. 3. Genes that were significantly differentially expressed between SCZ and CTL (*p* < 0.05) and had a persistence score > 0.5 or < − 0.5 were determined to have high or low persistence, respectively. This 0.5 cutoff marked genes with good separation from the rest of the dataset, and therefore suggests that these genes have especially meaningful persistence. At this cutoff, we identified 30 high persistence genes (4 in Deep, 15 in DISC1, and 11 in Super) and 7 low persistence genes (0 in Deep, 5 in DISC1, and 2 in Super) across our SCZ datasets (Table 1). We did not find much direct overlap (3 shared high persistence and 0 shared low persistence), likely due to the varied characteristics of these datasets.

**Table 1:** “Persistent” genes in SCZ datasets.

Deep				DISC1				Super			
High persistence		Low persistence		High persistence		Low persistence		High persistence		Low persistence	
Gene	Score	Gene	Score	Gene	Score	Gene	Score	Gene	Score	Gene	Score
TNR	0.663			CRYM	0.870	ALDH1A1	− 0.567	NEFM	0.870	UQCRB	− 0.594
ME3	0.625			GPD1	0.791	CLYBL	− 0.594	MBP	0.791	CORO2A	− 0.631
HIBADH	0.600			MBP	0.750	CHL1	− 0.631	TUBB2A	0.750		
CAPZA1	0.574			TNR	0.698	NRN1	− 0.875	NEFL	0.698		
				SYNPR	0.670	ATP1A4	− 0.960	ATP1B1	0.631		
				NCAN	0.663			INA	0.625		
				SLC32A1	0.631			HSPD1	0.600		
				KCNA1	0.625			GNAO1	0.592		
				SLC13A5	0.609			SH3BGRL2	0.574		
				NRGN	0.600			ATP6V0A1	0.561		
				BDH1	0.592			SEPT7	0.512		
				ME3	0.574						
				ACTN2	0.561						
				PLCXD3	0.525						
				GNG4	0.512						

Genes that are significantly differentially expressed in SCZ vs CTL and have high (> 0.5) or low (< − 0.5) persistence scores.

**Figure 4:**
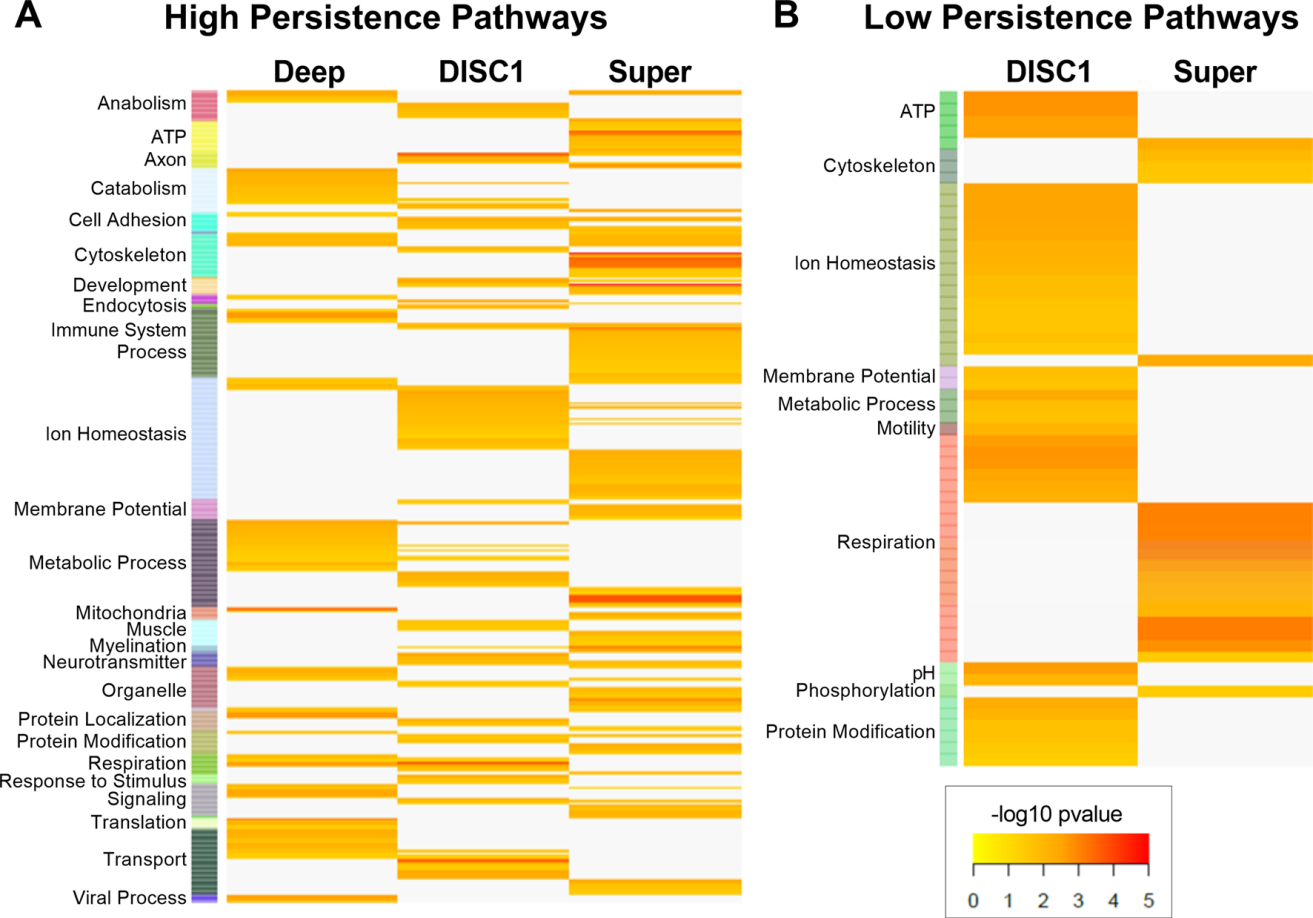
Persistent pathways in SCZ. Heatmaps of significant (*p* < 0.05) pathways associated with (**A**) high and (**B**) low persistence genes. Pathways have been assigned to functional categories, summarizing their major functions and indicating processes that are expected to be of high or low importance in SCZ, respectively. Figures produced using gplots^49^.

We then used Enrichr to run a pathway analysis on the high and low persistence genes identified in each dataset to gain deeper function insight^14^. Each set of genes was analyzed for significant (*p* < 0.05) enrichment in the gene ontology biological pathway, molecular function, and cellular component categories. Overall, we found 412 high persistence pathways (113 in Deep, 126 in DISC1, 149 in Super) and 59 low persistence pathways (0 in Deep, 39 in DISC1, 20 in Super). These pathways were further organized by functional themes to aid in meaningful interpretation (Fig. 4; Supplemental Table 2). The predominant theme in the high persistence genes was ion homeostasis, while the predominant low persistence theme was respiration. Other high persistence themes include metabolic process, immune system process, and transport; while other low persistence themes included ion homeostasis, ATP, and protein modification. Based on literature, we know that many of these persistent themes are known to play important roles in SCZ^15,16^, demonstrating the ability of persistence analysis to reveal meaningful functional results about disease states.

### Persistence improves RNA prediction of protein fold change

**Figure 5:**
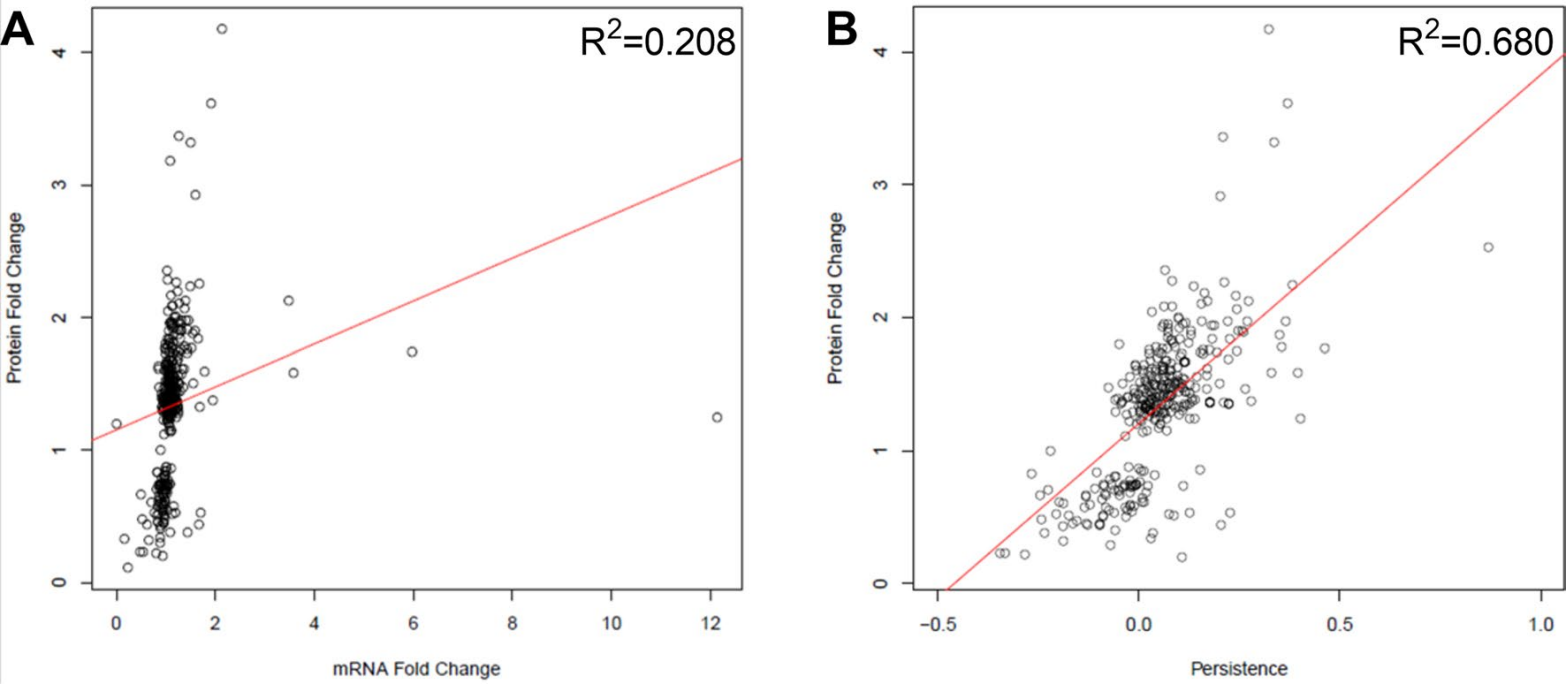
Persistence better predicts protein fold change in SCZ than mRNA fold change alone in the DISC1 dataset. (**A**) Correlation between protein and mRNA fold change (R^2^ = 0.208). (**B**) Correlation between protein fold change and persistence scores (R^2^ = 0.68). This improved correlation suggests that persistence can be predictive of protein abundance but room for improvement exists in the calculation. Figures produced using R base graphics^45^.

While the present persistence calculation appears to reveal meaningful insights into SCZ, we can compare our persistence scores to actual protein abundance measures to further demonstrate the value of this approach. While persistence should theoretically serve as a proxy for protein abundance, directly comparing the two will allow us to better define this relationship. The DISC1 dataset enabled us to make this comparison, as it contains information on both RNA and protein fold changes. While persistence did not generate a perfect correlation with protein fold change (Pearson Correlation R^2^ = 0.65), it did generate a stronger correlation with protein fold change than mRNA fold change alone (Pearson Correlation R^2^ = 0.208) (Fig. 5). This comparison suggests that persistence succeeds in improving our ability to predict protein abundance from RNA expression. While there is still room for improvement, the persistence calculation presented here represents a good starting point for improving our ability to estimate changes in protein from changes in RNA.

## Discussion

Persistence is a novel concept intended to expand the lens of transcriptomics to infer protein function by considering RNA expression and protein turnover together. Our goal is to address a common problems with transcriptomics, in that it tries to gain functional insight into protein abundance that is often missed by simply analyzing RNA expression^6–8^. We propose that one can approximate protein abundance by considering RNA expression in conjunction with how long the corresponding protein will last in the cell once it is translated (turnover ratio)^11,12^. While there is still room for improvement to make this link more complete between RNA and protein, we have shown that persistence is an insightful measure and that further work with the concept could be a valuable resource for computational biologists.

The lack of relationship between protein turnover and RNA expression suggests that a more complex metric is required to extract meaningful insights from the data (Fig. 1A–C). We also did not observe a particular enrichment of high or low turnover genes in SCZ relative to CTL (Fig. 1D). This suggests that there is not one specific type of turnover impacted by SCZ, but does not rule out that enrichment of certain high or low persistence genes happens in the disease. For these reasons, we developed the concept of persistence (Fig. 2) and demonstrated how it may be applied in 3 SCZ RNA datasets (1 RNAseq and 2 microarray).

In this analysis, we identified high and low persistence genes by selecting significantly differentially expressed genes with a persistence score > 0.5 or < − 0.5, respectively (Fig. 3; Table 1). These criteria yielded a small number of persistent genes that largely corresponded to pathways known to be altered in SCZ (Fig. 4). These results are encouraging that persistence is a meaningful measure because these pathways have been implicated in SCZ in prior studies. For example, ion homeostasis was the most commonly altered category in our high persistent pathways^17–22^. Specifically, potassium and sodium metal ion activity and transport appear to be more persistent in SCZ. Multiple studies in animals, iPSCs, and humans have noted increased potassium and sodium channel expression and activity in the prefrontal cortex. These changes were associated with abnormal neuronal activity, diminished synaptic plasticity, and impaired white matter integrity, all of which are characteristic of SCZ^17–22^. Additionally, antipsychotics can reverse these ion channel alterations, suggesting that this is a key mechanism in SCZ pathology^23–25^. Beyond ions, we also saw increased persistence in inflammatory system processes, which is consistent with observations of increased cytokine expression and immune system responsivity in SCZ^20,26–28^.

In terms of low persistence, we observed a decrease in respiration in SCZ. Specifically, there was low persistence in pathways associated with oxidative phosphorylation, suggesting that neurons in SCZ struggle to maintain sufficient levels of ATP production via aerobic respiration. Impaired oxidative phosphorylation and abnormal mitochondrial function has been noted in multiple SCZ studies^29–32^. This again indicates that there is a loss of efficiency in SCZ that puts more stress on the system and forces neurons to turn to alternate sources of energy. Indeed, postmortem samples from SCZ patients show an increase in lactate metabolism^29,33^. Interestingly, we also observed high persistence in metabolic processes, which further supports this concept of metabolic compensation for a loss of oxidative phosphorylation in SCZ. Overall, the genes and pathways identified by our persistence analysis are altered in SCZ, supporting the potential of this technique to extract important functional information from transcriptomics datasets.

The present persistence results did correlate fairly strongly with protein abundance (R^2^ = 0.68), which was a substantial improvement over RNA expression alone (R^2^ = 0.208). However, continued efforts to improve the persistence calculation are warranted (Fig. 5). We were only able to do this comparison in one dataset, as matching postmortem or iPSC tissue RNAseq and proteomics is rare, but this suggests that there is room for improvement in our persistence calculation. The present study was also limited by the narrow scope of the available turnover ratios. We only had ratios for ~ 2200 proteins, and these ratios were specific to synaptosomes. This left us with a narrow window into RNA datasets that needed to be specifically from neurons^34^. Of our more than 30 SCZ datasets, only 3 matched these criteria. With limited RNAseq/microarray datasets and turnover ratios, the present calculation is a good concept demonstration, but we would like to develop the concept of persistence further using expanded datasets and machine learning.

Ideally, this expansion would feature well-powered studies that have RNAseq and proteomics run on the same samples. This would allow us to use machine learning to integrate RNA expression and protein turnover ratios in the way that would most accurately predict protein expression in the same tissue^35,36^. A more complex relationship undoubtedly exists between these factors, so training a model to more accurately predict this relationship would be highly beneficial^10–12^. It would also be useful to expand the number and variety of protein turnover ratios to put into this model. This would require more SILAM studies^37,38^, but gathering this information from more animals in more tissues would greatly expand the context in which persistence could be applied. For example, another tissue for which turnover data is currently available is the liver^13^. We could use RNAseq data and liver cell protein turnover ratios to create models to predict protein abundance in a normally functioning liver. This model could then be used with RNAseq data from liver cancer to better identify the functional changes that occur in that disease state. While this would require time and money, it would add a new dimension to transcriptomics studies and improve our ability to understand mechanisms underlying various disorders. Especially high or low persistence genes may also represent important therapeutic targets since they will likely have a magnified role in disease mechanisms^39–41^.

Overall, we have demonstrated that applying protein turnover ratio data to RNA expression data represents a novel form of analysis that expands the amount of information that can be obtained from transcriptomics. Persistence combines RNAseq with protein turnover ratios to infer protein abundance, which is a more accurate measure of function in a cell. While the present study is limited by a lack of sufficient input data, it does identify high and low persistent genes and pathways that have been implicated in SCZ. Further development of the concept of persistence with expanded studies and machine learning techniques could greatly improve our ability to understanding of the molecular landscape in disease with RNAseq alone.

## Methods

### Turnover ratio and SCZ omics datasets

Turnover ratios for 2272 proteins were collected from Heo et al.^13^. Given that these turnover ratios were derived from synaptosomes, the present analysis was restricted to RNA datasets from neuronal populations alone. While this concept could be applied to a variety of subjects, we selected SCZ as our present focus, as it is a particularly pervasive disorder known to have widespread effects throughout the brain and a major focus in our laboratory. With these criteria in mind, we selected three neuronal, SCZ RNA datasets: Deep, DISC1, and Super^34^. The Deep and Super datasets were derived from pyramidal neurons cut from the dorsolateral prefrontal cortex of postmortem SCZ and CTL samples using laser capture microscopy. Specifically, the Deep dataset comes from the deep layers (IV-VI) of this region while the Super dataset comes from the superficial layers (I–III). Both of these datasets were obtained from RNA microarrays. The DISC1 dataset was derived from RNAseq run on induced pluripotent stem cells (iPSCs) obtained from SCZ patients positive for a DISC1 mutation and CTL siblings lacking the mutation. These iPSCs were differentiated into neurons and used for RNAseq analysis. Additionally, the DISC1 dataset included parallel proteomic data (mass spectroscopy) which allowed us to compare mRNA and protein. All datasets used in these analyses have previously been published and deposited in community-endorsed public repositories. Proteomics data used to derive protein turnover ratios^13^ were deposited at PXD007156. DISC1 RNAseq data^42^ were deposited at GSE57821. DISC1 proteomics data^43^ were deposited at PXD013391. Deep and Super microarray data^44^ were deposited at GSE145554.

### Investigating the relationship between protein turnover ratios and RNA expression in control and disease conditions

Regression analyses were performed to determine if proteins’ turnover ratios have a direct impact on the amount of corresponding RNA expression, or vice versa. If a significant relationship was identified, this would indicate that these two variables consistently impact each other and would make it easier to infer protein expression from RNA expression. Scatterplots of protein turnover versus mRNA expression we generated utilizing R base graphics^45^ for the Deep, DISC1, and Super datasets (Fig. 1A–C). A linear regression model was computed for both groups of data using the R base statistics *lm* function^45^. This was performed for both CTL and SCZ data to determine if disease state impacts this relationship.

Furthermore, we investigated if SCZ selectively impacts particular subsets of proteins based upon their turnover ratio. It is possible that a disease could have a greater impact on longer versus shorter lived proteins, or vice versa, even in the absence of an overall relationship. For this analysis, we generated a turnover distribution in which genes were sorted into bins of low to high turnover ratios: < 2, 2–3, 3–4, 4–5, and > 5 respectively. Differentially expressed genes (DEGs) (*p* < 0.05) identified in the three SCZ datasets were overlaid with these bins to determine the number of genes in each turnover bin (Fig. 1D). A one-way ANOVA was used to determine if there was any significant difference between the numbers of DEGs in each bin, which would be suggestive of a particular bin being more strongly affected by SCZ. This analysis was performed in GraphPad Prism 8^46^.

### Generating the concept of “persistence”

While direct, predictable relationships do not exist between RNA expression, protein turnover, and protein expression, we posited that it may be possible to combine the former two factors to more successfully predict the latter. The RNA present in a cell will be translated into protein that will remain in that cell for a given amount of time. It stands to reason that genes with more RNA and less turnover would generate proteins that were more abundant and long-lasting. These proteins would therefore “persist” in that cell more, lending them a greater opportunity to have functional impact. On the other hand, genes with less RNA and faster turnover would “persist” less and would be expected to have a lesser functional impact (Fig. 2A). Based upon this concept, we generated the concept of persistence, which utilizes the Huganir turnover ratios^13^ and RNA fold change to generate a measurement of the potential functional impact of changes in RNA expression. The persistence calculation was conducted as follows:

1 $$Persistence = \frac{RNA \;log2\;Fold\;Change}{{Turnover\;Ratio}}.$$

This equation is designed to generate a persistence score in which high values indicate high persistence, meaning that that particular gene is produced more in the disease state (high RNA fold change) and stays around longer to exert more activity (low turnover ratio [i.e. long half-life]). On the other hand, low persistence suggests that genes are produced less (low RNA fold change) and are quickly degraded (high turnover ratio [i.e. short half-life]), resulting in a lower overall impact in the synaptosome. Theoretically, this combination of RNA fold change and turnover ratio should act as a proxy for protein fold change. R code for calculating persistence can be found in Supplemental Code 1, and an outline of this script is provided in Fig. 1B. Input files to demonstrate the use of this code and serve as a template for other users are provided as Supplemental Code Input 1–4.

### Identifying persistent genes in SCZ

Persistence scores were calculated for the Deep, DISC1, and Super datasets and quantile normalized using the *preprocessCore* library in R to allow for comparisons across datasets^47^. In order to call a gene “persistent,” it (1) needed to be significantly changed in SCZ versus CTL (*p* < 0.05) and (2) have a persistence score greater than 0.5 (high persistence) or less than − 0.5 (low persistence). Genes that met both of these criteria are listed in Table 1, and the distribution of significance and persistence scores is presented as a volcano plot for each dataset (Fig. 3). The volcano plots were then generated using the *ggplot2* R package^48^ with persistence on the x-axis and -log10 p-value on the y-axis. Genes that met the significance criteria were colored in red and those that further met the persistence criteria are labeled with their gene names.

### Functional themes associated with persistent genes in SCZ

The significant, persistent genes identified above were then run through pathway enrichment analysis to determine their functional associations. Pathway analysis was performed utilizing Enrichr^14^, specifically the three Gene Ontology databases: Cellular Component, Biological Process, and Molecular Function. The analysis was performed for the set of genes with high and low persistence separately, yielding a set of enriched pathways for each. These results are presented as unionized heatmaps which display the significance (− log10 *p* values) of different pathways across the persistent genes in the Deep, DISC1, and Super datasets (Fig. 4). To aid in interpretation of the functional implications of these results, individual pathway annotations were categorized into themes using a priori knowledge. Heatmaps were generated using the library *gplots*^49^ and GraphPad Prism 8^46^. Pathways and themes that replicated across datasets were of higher confidence, and therefore more likely to really have high or low persistence in SCZ. A review of SCZ literature was used to further support the validity of these results.

### Persistence as a proxy for protein expression

While we expect that persistence should increase our ability to infer protein expression from RNA expression and protein turnover, we were able to directly test this concept using the DISC1 dataset, as it contains transcriptomics and proteomics data from the same tissue. Regression analyses were used to determine the relationship between (1) RNA and protein expression and between (2) persistence scores and protein expression (Fig. 5). The former determines the ability of RNA expression alone to predict protein expression, which as we know from the literature should be poor. The latter determines the ability of persistence (considering RNA expression and protein turnover ratios together) to predict protein expression. An increased R^2^ between these analyses would suggest that persistence improves our ability to infer protein expression, and thereby more functionally relevant information, from transcriptomics data. Specifically, R base graphics were used to generate a (1) mRNA log2 fold change versus protein log2 fold change scatterplot for the DISC1 dataset and (2) persistence versus protein log2 fold change scatterplot. Correlations were computed using R base statistics and a linear regression model was fitted using R base statistics^45^.

## Supplementary Information

Supplementary Information 1.

Supplementary Information 2.

Supplementary Information 3.

Supplementary Information 4.

Supplementary Information 5.

Supplementary Information 6.

Supplementary Information 7.

Supplementary Information 8.

## Data Availability

The datasets analyzed during the current study are available in the NCBI Gene Expression Omnibus repository (GSE57821 and GSE145554) and the ProteomeXchange Consortium via the PRIDE partner repository (PXD007156 and PXD013391).

## Code availability

R code for persistence is provided in Supplemental Code 1. This code can be used to calculate persistence scores and identify genes of high and low persistence in any input transcriptomics dataset. Additional code that was used to generate the findings of this study is available from the corresponding author, upon reasonable request.
