# Supplementary material for: Using protein turnover to expand the applications of transcriptomics
Source: Sci Rep. 2021 Feb 23;11:4403. doi: 10.1038/s41598-021-83886-7 (PMC7902815; doi:10.1038/s41598-021-83886-7)
Supplement: Supplementary file 8 — Supplementary Information 8. [file 41598_2021_83886_MOESM8_ESM.docx]

**Using Protein Turnover to Expand the Applications of Transcriptomics**

Marissa A. Smail^1,2^^*, James K. Reigle^3^^, Robert E. McCullumsmith^4^

Author Affiliations

^1^Department of Pharmacology and Systems Physiology, University of Cincinnati, Cincinnati, OH, United States

^2^Neuroscience Graduate Program, University of Cincinnati, Cincinnati, OH, United States

^3^Biomedical Informatics, Cincinnati Children’s Hospital Medical Center, Cincinnati, OH, United States

^4^Department of Neurosciences, University of Toledo College of Medicine and Life Sciences, Toledo, OH, United States

^Co-first authors

*Corresponding author:

Marissa A. Smail

University of Cincinnati,

Department of Pharmacology and Systems Physiology

2170 E. Galbraith Rd. Bldg E. Room 216

Cincinnati, OH 45237-0506

United States

smailma@mail.uc.edu

SUPPORTING INFORMATION LEGENDS

**Supplemental Table 1: Persistence information for full SCZ datasets.** Persistence scores, -log 10 p values, and significance information for 2101 genes in Deep, DISC1, and Super dataset.

**Supplemental Table 2: Full pathway information from Figure 4 heatmaps.** Details regarding pathways associated with (A) high and (B) low persistence genes.

**Supplemental Code 1: Generating persistence scores and identifying persistent genes from transcriptomics data.** Code that can input any transcriptomics dataset with log2 fold changes and pvalues and generate persistence information as described in the manuscript. See annotations in code for running the analysis. Files for running the code are provided as the supplemental code inputs listed below.

**Supplemental Code Input 1: Protein Turnover Input File.** Protein turnover ratios used in persistence calculation. Input into Supplemental Code 1 at line 30.

**Supplemental Code Input 2: “Super” Transcriptomics Input File.** Transcriptomics input file for Super dataset. Provided as a demonstration of the persistence calculation and template for other transcriptomics datasets with log2FC and pvalues that can be analyzed using this script. Input into Supplemental Code 1 at line 11. Note that the 3 datasets were quantile normalized prior to other analyses, so raw values output here may not match those presented in the rest of the manuscript.

**Supplemental Code Input 3: “Deep” Transcriptomics Input File.** Transcriptomics input file for Deep dataset. Alternative input into Supplemental Code 1 at line 11 (change line to “Supplemental Code Input 3”).

**Supplemental Code Input 4: “DISC1” Transcriptomics Input File.** Transcriptomics input file for DISC1 dataset. Alternative input into Supplemental Code 1 at line 11 (change line to “Supplemental Code Input 4”).
